# Intraoperative electron radiation therapy (IOERT) in patients with locally recurrent renal cell carcinoma

**DOI:** 10.1186/1748-717X-8-282

**Published:** 2013-12-02

**Authors:** Gregor Habl, Matthias Uhl, Frank Hensley, Sascha Pahernik, Juergen Debus, Falk Röder

**Affiliations:** 1 Department of Radiation Oncology, University of Heidelberg, Im Neuenheimer Feld 400 Heidelberg 69120, Germany; 2 Department of Urology, University of Heidelberg, Heidelberg, Germany

**Keywords:** IOERT, IORT, Recurrent renal cell carcinoma, Renal cell carcinoma, Renal fossa

## Abstract

**Background:**

To analyze our experience with intraoperative electron radiation therapy (IOERT) followed by moderate doses of external beam radiation therapy (EBRT) in patients with locally recurrent renal cell carcinoma.

**Methods:**

From 1992 to 2010, 17 patients with histologically proven, locally recurrent renal cell carcinoma (median tumor size 7 cm) were treated by surgery and IOERT with a median dose of 15 Gy. All patients met the premise of curative intent including 7 patients with oligometastases at the time of recurrent surgery, which were resected and/or irradiated. The median time interval from primary surgery to local recurrence was 26 months. Eleven patients received additional 3D-conformal EBRT with a median dose of 40 Gy.

**Results:**

Surgery resulted in free but close margins in 6 patients (R0), while 9 patients suffered from microscopic (R1) and 2 patients from macroscopic (R2) residual disease. After a median follow-up of 18 months, two local recurrences were observed, resulting in an actuarial 2-year local control rate of 91%. Eight patients developed distant failures, predominantly to liver and bone, resulting in an actuarial 2-year progression free survival of 32%. An improved PFS rate was found in patients with a larger time interval between initial surgery and recurrence (> 26 months). The actuarial 2-year overall survival rate was 73%. Lower histological grading (G1/2) was the only factor associated with improved overall survival. Perioperative complications were found in 4 patients. No IOERT specific late toxicities were observed.

**Conclusions:**

Combination of surgery, IOERT and EBRT resulted in high local control rates with low toxicity in patients with locally recurrent renal cell cancer despite an unfavorable surgical outcome in the majority of patients. However, progression-free and overall survival were still limited due to a high distant failure rate, indicating the need for intensified systemic treatment especially in patients with high tumor grading and short interval to recurrence.

## Background

Renal cell carcinoma (RCC) is the most common type of kidney cancer in adults [1]. Surgery represents the cornerstone of approaches with curative intent treatment and even plays a role in primary metastatic disease [2]. However, even in patients receiving curative surgery for localized disease at presentation, overall survival is limited, mainly due to the high rates of early distant rather than local failures. Therefore the main recent research efforts focused on the development of adjuvant systemic therapies rather than intensification of local treatment for example by adjuvant irradiation, which showed no survival benefit according to randomized trials during the 1970s and 80s [3-5].

However, a small fraction of patients reported to be in the range of 0-17% depending on initial tumor size [6], will develop an isolated local recurrence without evidence of distant spread after curative surgery. As these patients have obviously not developed early metastatic spread, they reasonably represent a patient group for a salvage treatment approach with curative intent.

In contrast to primary disease, local recurrences of renal cell carcinomas share some features with other tumor entities located in the renal fossa, especially soft tissue sarcoma [7]. Both often present as large tumor masses directly adjacent to vital structures or the abdominal wall, and thus limit the surgical ability to achieve wide resection margins. However, resection margin has been reported as the most important prognostic factor for local control and survival not only for retroperitoneal sarcoma, but also in patients experiencing an isolated local recurrence of renal cell cancer [8]. Therefore it seems reasonable to consider similar additional local therapies like radiation therapy for both patients groups. Unfortunately, they also share the feature of low radiation sensitivity while being surrounded by organs at risk with low radiation tolerance like stomach, small bowel, contralateral kidney, liver and spinal cord. Using external beam radiation alone would lead to dose limitations with restricted efficacy or considerable toxicity, especially if conventional radiation techniques are used [5].

Intraoperative electron radiation (IOERT) is a technique which includes the possibility to surgically displace organs at risk with low radiation tolerance from the target volume while applying a large single dose to the regions at high risk for incomplete resection. Thus, IOERT offers the possibility to overcome these dose limitations, especially if combined with moderate doses of postoperative external beam radiation therapy. Therefore IOERT has been introduced in the treatment of retroperitoneal tumors at our center more than two decades ago and has been shown to result in increased local control at least for sarcoma patients by our and other research groups [7,9].

For these reasons, we offered our patients with locally recurrent renal cell carcinoma a similar multimodality treatment approach. It consisted of maximal surgery and IOERT followed by moderate doses of postoperative EBRT in order to avoid severe radiation side effects to abdominal organs at risk while escalating the dose to the tumor bed to improve local control.

In this work we present our experience using this approach based on a retrospective evaluation.

## Methods

Between 1992 and 2010, 17 patients with histologically proven recurrent renal cell carcinoma were treated in our institution with surgery and IOERT boost +/- postoperative EBRT. All recurrences were located in the renal fossa. The median time interval from primary surgery to recurrence was 26 month (2–115 months). All patients were restaged with at least abdominal CT or MRI and x-rays of the lung. Further imaging to exclude distant metastases took place in case of clinical suspicion. Patients were selected for this multimodality approach by a surgeon and radiation oncologist. Indication for IOERT was seen if complete surgical removal with microscopic free margins seemed impossible or at least questionable, although gross complete resection was attempted in all cases. Seven of the 17 patients showed limited metastatic disease, which was attempted for resection and/or irradiation. Hence, all patients met the premise of curative intent treatment. Further patient characteristics are shown in Table 1.

**Table 1:** Lesion and treatment characteristics

	**n**	**%**		**n**	**%**
**Age**			**EBRT**		
Median	61		Yes	11	65
Range	32-76		No	6	35
**Gender**			**EBRT dose**		
Male	11	65	Median	40	
Female	6	35	Range	36-43,2	
**Time to rec.**			**Adj. CHT**		
Median	26		Yes	2	12
Range	2-115		No	15	88
**Histology**			**Resection margin**		
Clear cell	14	82	R0	6	35
Papillary	2	12	R1	9	53
Sarcomatoid	1	6	R2	2	12
**Tumor size**			**IORT dose**		
Median	7		Median	15	
Range	3-14		Range	10-20	
**Grading**			**IORT energy**		
G1/2	13	76	Median	8	
G3	4	24	Range	6-12	
Distant met.*			**IORT cone**		
Yes	7	41	Median	10	
No	10	59	Range	6-18	

Age: [years], Time to rec. : Time from surgery of primary lesion until surgery for recurrent lesion [months], Tumor Size: [cm], Distant Met: History of distant metastasis prior to or at actual treatment, EBRT: External Beam Radiation Therapy, IORT: Intraoperative Radiation Therapy, EBRT/IORT dose: [Gy], adj CHT : adjuvant Chemotherapy (actual treatment), IORT Energy: [MeV], IORT cone: [cm].

IOERT was performed in a dedicated operating room with an integrated Siemens Mevatron ME linear accelerator (Siemens, Concord, CA). The IOERT dose was prescribed to the 90% isodose level and the IOERT field was selected to cover the area of suspected microscopic residual disease. After defining the target area in coordination with the surgeon, an applicator with appropriate diameter was chosen, manually positioned and attached to the table. Lead shields of 5 mm thickness could be used to protect or displace uninvolved tissue. The patient was then located beneath the linear accelerator and the applicator was aligned by a laser air-docking system at a focus-to-surface distance of 100 cm. The electron energy was selected according to the depth of tissue to be irradiated. The median IOERT dose was 15 Gy, using a median electron energy of 8 MeV, with a median applicator size of 10 cm. Dose prescription was based on surgeons assessment of margin status including increasing but not routine use of intraoperative pathologic assessment of frozen sections during the overall study period. In general, higher IOERT doses were applied in cases suspicious of positive margins or residual disease according to the. surgeons assessment. In 11 patients, additional postoperative 3D-conformal EBRT was applied with a median dose of 40 Gy in conventional fractionation. Six patients received no EBRT due to postoperative complications or patient´s refusal. Only 2 patients received adjuvant systemic therapy. For detailed treatment characteristics see Table 1.

Follow-up examinations took place in our institution and/or the Department of Urology. Patients lost to routine follow-up were contacted by phone. Local recurrence was defined as tumor growth in the renal fossa. Progression-free survival was defined as absence of local/distant failure or death from any cause. Overall survival was defined as absence of death from any cause. Toxicities from surgery, IOERT and EBRT were pooled because of the difficulty to precisely distinguish between the toxicity caused by each separate treatment. Descriptive statistics and Kaplan-Meier estimation analysis were applied for statistical workup. The 1- and 2-year actuarial rates for local control, progression-free survival and overall survival for the entire group and various subgroups were calculated. Statistical differences in time-to-event data were tested by the log rank test. Relations between distinct parameters were tested for significance by Fisher´s exact test. Differences were considered statistically significant for a p-value of ≤ 0.05. The study is in compliance with the Declaration of Helsinki (Sixth Revision, 2008). Furthermore, the study was approved by the Independent Ethics committee of the Medical Faculty at the University of Heidelberg.

## Results

Complete surgical removal with free margins (R0) was achieved in only six patients. Nine patients showed involved margins (R1) and in two patients gross residual disease (R2) was present. Subgroup analyses revealed statistically significant associations between tumor size < 7 cm (p = 0,035) or resection of adjacent organs (p = 0,035) with an increased probability of microscopic complete resections.

**Figure 1:**
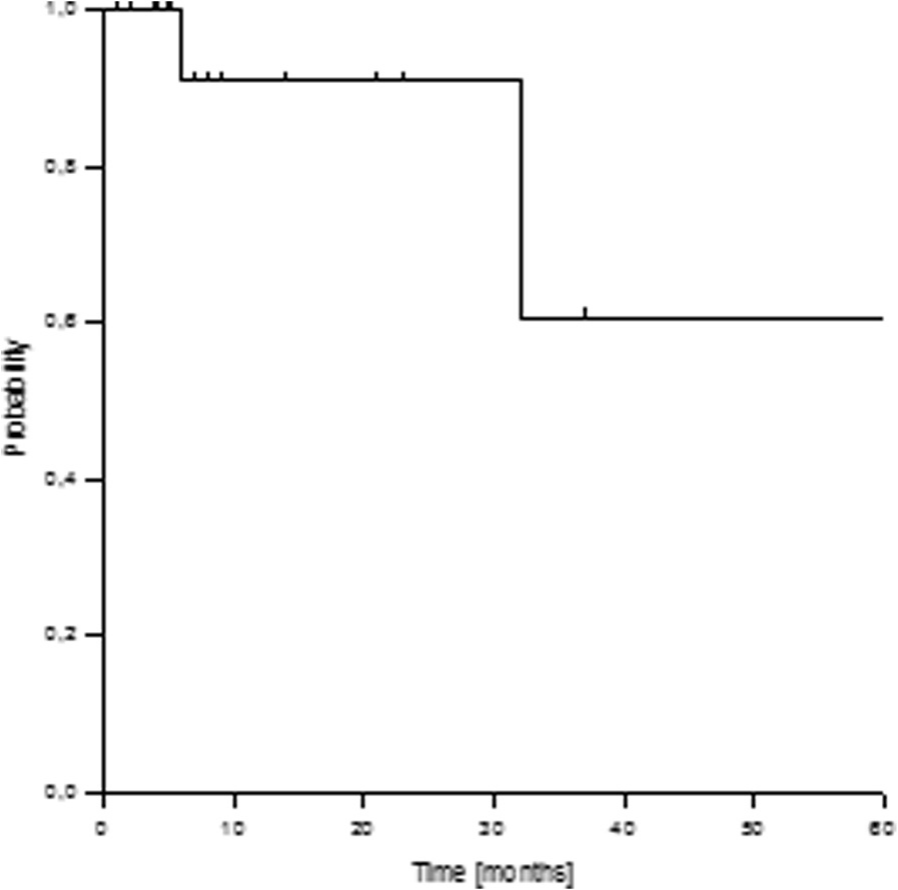
Actuarial local control rate.

After a median follow-up of 18 months (2–211 months), two local recurrences were observed in the entire group, resulting in actuarial 1- and 2-year local control rates of 91%, respectively (Figure 1). The recurrences were classified as one infield-IOERT and one outfield-EBRT field recurrence. No factors could be established as significantly predictive for local control in the subgroup analyses.

Interestingly, two of the seven patients with limited distant metastases at time of recurrent surgery were among the three patients with survival times of more than 15 years, indicating that long-term survival is possible if adequate local control is achieved even in patients with unfavorable disease situations.

**Figure 2:**
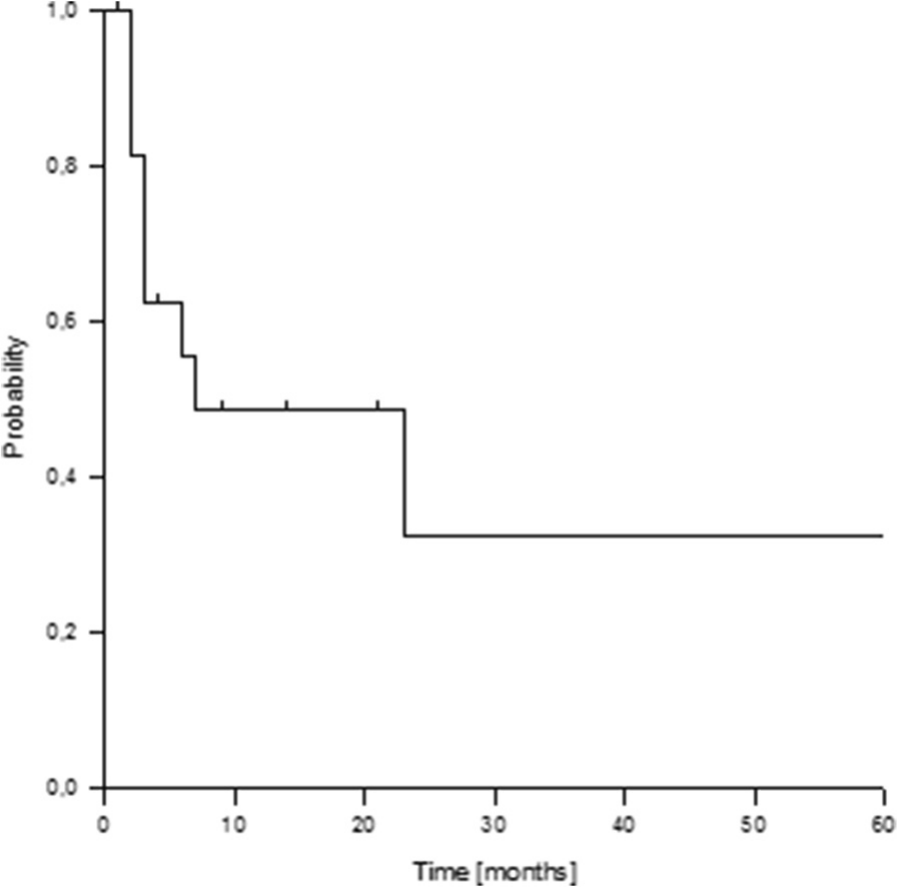
Actuarial progression free survival rate.

Eight patients developed new distant metastasis, mainly to liver (29%) and bone (24%). The resulting actuarial 1- and 2-year progression free survival rate for the entire group was 49% and 32% (Figure 2). Univariate subgroup analyses revealed a significantly improved progression free survival rate in patients with a time interval between initial surgery and recurrence >26 months (p = 0.015).

**Figure 3:**
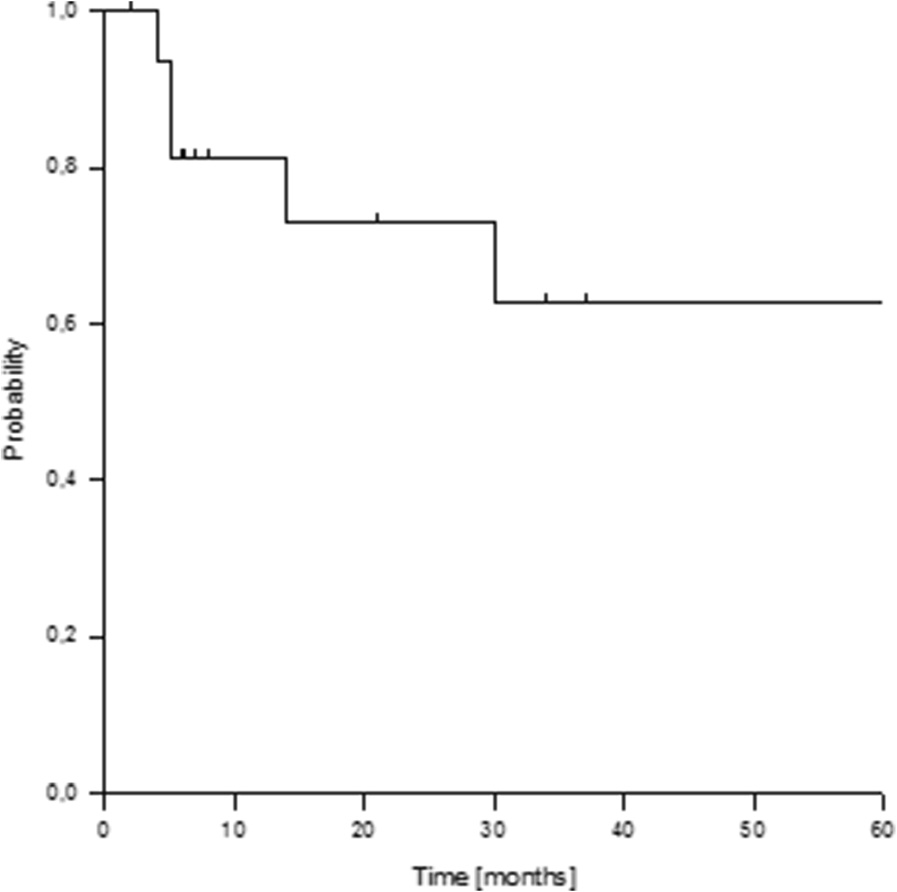
Overall survival.

The actuarial 1- and 2-year overall survival rates were 81% and 73%, respectively (Figure 3). Lower histological grade (G1/2 vs G3) was the only factor which significantly correlated with improved overall survival (p = 0,003) according to univariate subgroup analyses.

Perioperative toxicity was seen in four patients, particularly as wound healing disturbance in one patient and intraabdominal abscesses in two patients. One patient developed acute renal failure and hyperglycemia. No severe acute or late toxicities specifically attributable to IOERT or EBRT were observed.

## Discussion

Treatment approaches with curative intent for renal cell carcinoma in primary situation mostly consist of surgery with or without adjuvant systemic therapy due to the considerable risk for distant failure, which still represents the limiting factor for improved overall survival. Radiation therapy is usually not part of the primary treatment, because the risk of isolated local recurrences seems much lower than the risk of distant spread and sufficient radiation doses are hardly achievable with conventional techniques because of the low tolerance of the surrounding organs at risk. Consequently, randomised trials evaluating adjuvant radiation therapy during the 1970s and 80s failed to show a survival benefit in comparison to surgery alone [3-5], thus limiting the role of radiation therapy mainly to palliative situations.

However, a small patient group will develop an isolated local recurrence after curative intent surgery. As these patients did not suffer from early distant spread but failed locally, one may argue in favor of a specific disease stage with a different tumor biology, which justifies a salvage treatment approach with curative intent. Although data regarding this situation are rare, surgery alone will frequently result in microscopic or gross positive margins given the often advanced lesion sizes combined with the unfavorable localization [10,11]. Even after complete resection, these patients will be at high risk for local re-recurrence [12], since wide margins seem hard to achieve, thus leading to the rationale for additional radiation therapy.

However, as addressed earlier, sufficient radiation doses to control residual disease seem hardly achievable using external radiation therapy techniques alone, due to the limited radiation tolerance of adjacent organs at risk. Therefore several groups including ours have proposed the introduction of IOERT into the treatment strategy in order to escalate the dose to the high risk regions without compromising the tolerance of the surrounding tissue, especially small bowel.

Using a combination of maximum surgery, IOERT and moderate doses of postoperative external beam radiation therapy, we observed a highly satisfactory estimated 2-year local control rate of 91%, especially regarding the unfavorable surgical outcome with microscopic or gross residual disease in the majority of our patients. Moreover, this approach was not accompanied by increased toxicity compared to surgery and EBRT alone [3-5].

These findings are in line with the reports of other groups using similar treatment approaches including IOERT (see Table 2). For example, investigators from the University of Pamplona reported early outcome data on 11 patients suffering from locally advanced primary or recurrent renal cell carcinoma, treated with surgery and IOERT [13]. Seven patients received additional external beam radiation therapy with 30–45 Gy. They observed only one local recurrence and found no additional toxicity due to IOERT in short-term follow-up. At the University of San Francisco, ten patients with locally recurrent renal cell cancer have been treated with surgery plus IOERT. Only two local recurrences were observed after a median follow up of 6 years [14]. The groups from Mayo [10] and Madrid-Pamplona [11] also reported crude local control rates in excess of 80% after mature follow up (more than 10 years) in the largest published series on the use of IOERT for locally advanced or recurrent renal cell cancer, thus indicating the long-term durability of local control. They also confirmed the low overall rates of severe toxicity (less than 20%) with this approach.

**Table 2:** Outcomes after surgery + IOERT +/- EBRT for primary/recurrent renal cell carcinoma

**Series**	**Primary/recurrent**	**f/u med (y)**	**Median IORT dose (Gy)**	**Median EBRT dose (Gy)**	**EBRT number**	**3-y OS (%)**	**Local control**
Pamplona [13]	8/3	0.7	15	30-45	7/15	Not stated	10/11
San Francisco [14]	0/10	5.9	15	none	0/10	36	8/10
Mayo [10]	3/19	9.9	13.5	48	21/22	50	17/22
Madrid-Pamplona (pooled) [11]	15/10	22.2	14	44	15/25	38	21/25
Current study	0/17	1.5	15	40	11/17	63	15/17

These figures compare well with surgery only series, although data on local control with surgery alone are extremely rare. To our knowledge, Margulis et al. [12] published the largest series reporting data on local control after treatment of local recurrences with surgery alone. After a median follow up of 41 months, they described a 15% rate of isolated local re-recurrences in their series of 54 patients. Another 50% developed distant failure with or without local failure, indicating an even higher local re-recurrence rate, although the overall incidence of local re-recurrences was not stated. The authors further identified a positive margin resection as a negative predictor of cause-specific survival in their series. Although not reported precisely, it can be estimated from their risk model, that less than one third of their patients had a positive margin compared to two thirds in our cohort. Given the unfavourable surgical outcome, it therefore seems likely that the addition of IOERT has contributed to the low incidence of local recurrences in our and other IOERT series with even higher rates of margin positive resections [10,11].

However, we observed considerable rates of distant metastases in our cohort, which compromised progression-free and overall survival. Our results seemed comparable to the findings of other groups using treatment approaches with [10,11,13-15] or without IOERT [12,16-19]. For example, Hallemeier et al. [10] reported 1- and 5-year PFS rates of 64% and 31%, which were mainly influenced by a high rate of distant failures in their series of patients treated with surgery and IOERT. Estimated OS-rates at 1 and 5 years were 91% and 40%, respectively. Calvo et al. [11] described 1- and 5-year PFS rates of 59% and 18% using a similar treatment approach. Again distant failure was the major pattern of relapse. Overall survival at 1 and 5 years was 71% and 38%. Margulis et al. [12] found a distant failure rate of 50% in their series using surgery alone with additional systemic therapy in 69% of the patients. Although not stated, 1- and 2-year PFS rates of about 45% and 32% can be estimated from their published Kaplan-Meier curves, which are similar to our findings. Nevertheless, they reported an impressive median cause-specific survival of 61 months, though this figure should be interpreted with caution since 15% of their patients died of non-renal cell cancer causes. Similarly Itano et al. [18] described a 5-year disease-specific survival of 51% in a subgroup of 11 patients treated by surgery alone. In contrast, only 4 of 11 patients survived for more than 2 years after surgery alone in the series of Esrig et al. [19]. Taken together, progression-free and overall survival in these patients seem to be mainly limited by distant control with or without IOERT, although keeping in mind, that such comparisons are likely biased by differences in patient selection. Nevertheless, this raises the question for additional systemic treatment, which has not been applied to the majority of our patients.

We identified a short time interval between primary surgery and recurrence and high histological grading (G3) as factors associated with poor survival, although these results should be interpreted with extreme caution since patient numbers were very small. Especially patients with the mentioned adverse features may benefit from early intensified systemic therapy and should therefore be excluded at least from the time-consuming postoperative external beam radiation phase.

Clearly, our study has some limitations, especially regarding its retrospective nature, the small patient number and the rather short follow up. Therefore conclusions should be drawn with caution. However, given the rarity of this specific disease situation, adequately powered prospective studies comparing different treatment schedules seem very unlikely. Therefore publication of all cases and treatment approaches seems valuable to give treating physicians as much information as possible for the treatment of these challenging patients. Further on, our data has been included into a multi-institutional pooled analysis led by the MGH group, which mainly confirms our findings in a larger patient group, but so far has been only published in abstract form [20]. We strongly encourage other groups to participate in similar efforts.

## Conclusion

In summary, our study represents one of the largest single center series evaluating the role of IOERT in the treatment of recurrent renal cell carcinoma. Combination of maximum surgery, IOERT and EBRT resulted in high local control rates with low toxicity despite an unfavorable surgical outcome in the majority of patients. However, progression-free and overall survival were still limited due to a high distant failure rate, indicating the need for intensified systemic treatment especially in patients with high tumor grading and short interval to recurrence.

## Competing interests

The authors declare that they have no competing interests.

## Authors’ contributions

GH participated in study design, data analysis, patient treatment and drafted the manuscript. MU, FH and SP participated in data analysis, manuscript draft and treatment of the patients. JD revised the manuscript critically. FR designed the study, participated in data analysis, patient treatment, manuscript draft and revised it critically. All authors read and approved the final manuscript.

## References

[B1] Rini BI Campbell SC Escudier B Renal cell carcinoma Lancet 2009 8 1119 1132 10.1016/S0140-6736(09)60229-4 19269025

[B2] Flanigan RC Mickisch G Sylvester R Tangen C Van Poppel H Crawford ED Cytoreductive nephrectomy in patients with metastatic renal cancer: a combined analysis J Urol 2004 8 1071 1076 10.1097/01.ju.0000110610.61545.ae 14767273

[B3] Finney R The value of radiotherapy in the treatment of hypernephroma–a clinical trial Br J Urol 1973 8 258 269 10.1111/j.1464-410X.1973.tb12152.x 4712834

[B4] Kjaer M Frederiksen PL Engelholm SA Postoperative radiotherapy in stage II and III renal adenocarcinoma. A randomized trial by the Copenhagen Renal Cancer Study Group Int J Radiat Oncol Biol Phys 1987 8 665 672 10.1016/0360-3016(87)90283-5 3553111

[B5] Fugitt RB Wu GS Martinelli LC An evaluation of postoperative radiotherapy in hypernephroma treatment–a clinical trial Cancer 1973 8 1332 1340 10.1002/1097-0142(197312)32:6<1332::AID-CNCR2820320607>3.0.CO;2-E 4757923

[B6] Morgan WR Zincke H Progression and survival after renal-conserving surgery for renal cell carcinoma: experience in 104 patients and extended followup J Urol 1990 8 852 857 2398558 10.1016/s0022-5347(17)39608-8

[B7] Roeder F Schulz-Ertner D Nikoghosyan AV Huber PE Edler L Habl G Krempien R Oertel S Saleh-Ebrahimi L Hensley FW Buechler MW Debus J Koch M Weitz J Bischof M A clinical phase I/II trial to investigate preoperative dose-escalated intensity-modulated radiation therapy (IMRT) and intraoperative radiation therapy (IORT) in patients with retroperitoneal soft tissue sarcoma BMC Cancer 2012 8 287 10.1186/1471-2407-12-287 22788989 PMC3495760

[B8] Rabinovitch RA Zelefsky MJ Gaynor JJ Fuks Z Patterns of failure following surgical resection of renal cell carcinoma: implications for adjuvant local and systemic therapy J Clin Oncol 1994 8 206 212 8270978 10.1200/JCO.1994.12.1.206

[B9] Roeder F Timke C Uhl M Habl G Hensley FW Buechler MW Krempien R Huber PE Debus J Werner J Aggressive local treatment containing intraoperative radiation therapy (IORT) for patients with isolated local recurrences of pancreatic cancer: a retrospective analysis BMC Cancer 2012 8 295 10.1186/1471-2407-12-295 22809267 PMC3414804

[B10] Hallemeier CL Choo R Davis BJ Pisansky TM Gunderson LL Leibovich BC Haddock MG Long-term outcomes after maximal surgical resection and intraoperative electron radiotherapy for locoregionally recurrent or locoregionally advanced primary renal cell carcinoma Int J Radiat Oncol Biol Phys 2012 8 1938 1943 10.1016/j.ijrobp.2011.02.026 21514065

[B11] Calvo FA Sole CV Martinez-Monge R Azinovic I Aristu J Zudaire J Garcia-Sabrido JL Berian JM Intraoperative EBRT and resection for renal cell carcinoma: twenty-year outcomes Strahlenther Onkol 2013 8 129 136 10.1007/s00066-012-0272-3 23223810

[B12] Margulis V McDonal M Tamboli P Swanson DA Wood GG Predictors of oncological outcome after resection of locally recurrent renal cell carcinoma J Urol 2009 8 2044 2051 10.1016/j.juro.2009.01.043 19286220

[B13] Santos M Ucar A Ramos H Escude L Berian JM Zudaire J Calvo FA Intraoperative radiotherapy in locally advanced carcinoma of the kidney: initial experience Actas Urol Esp 1989 8 36 40 2711906

[B14] Master VA Gottschalk AR Kane C Carroll PR Management of isolated renal fossa recurrence following radical nephrectomy J Urol 2005 8 473 477 10.1097/01.ju.0000165574.62188.d0 16006867

[B15] Eble MJ Stahler G Wannenmacher M IORT for locally advanced or recurrent renal cell carcinoma Front Radiat Ther Oncol 1997 8 253 255 9263835 10.1159/000061173

[B16] Gogus C Baltaci S Beduk Y Sahinli S Kupeli S Gogus O Isolated local recurrence of renal cell carcinoma after radical nephrectomy: experience with 10 cases Urology 2003 8 926 929 10.1016/S0090-4295(02)02582-7 12736006

[B17] Schrodter S Hakenberg OW Manseck A Leike S Wirth MP Outcome of surgical treatment of isolated local recurrence after radical nephrectomy for renal cell carcinoma J Urol 2002 8 1630 1633 10.1016/S0022-5347(05)65167-1 11912377

[B18] Itano NB Blute ML Spotts B Zincke H Outcome of isolated renal cell carcinoma fossa recurrence after nephrectomy J Urol 2000 8 322 325 10.1016/S0022-5347(05)67350-8 10893575

[B19] Esrig D Ahlering TE Lieskovsky G Skinner DG Experience with fossa recurrence of renal cell carcinoma J Urol 1992 8 1491 1494 1593672 10.1016/s0022-5347(17)37605-x

[B20] Paly JJ Hallemeier CL Biggs PJ Niemierko A Roeder F Martinez-Monge R Whitson JM Calvo FA Fastner G Efstathiou JA Outcomes for a multi-institutional cohort of patients treated with intraoperative radiation therapy for advanced or recurrent renal cell carcinoma [abstract] Int J Radiat Oncol Biol Phys 2012 8 S424 425 10.1016/j.ijrobp.2013.11.207 24411190

